# Early versus delayed mobilisation for non-surgically treated proximal humerus fractures: a systematic review and meta-analysis of randomised trials

**DOI:** 10.1186/s12891-025-08371-y

**Published:** 2025-02-27

**Authors:** Dimitris Challoumas, Haroon Minhas, Stephanie Bagni, Neal Millar

**Affiliations:** School of Infection and Immunity, College of Medical, Veterinary and Life Sciences, University of Glasgow, Glasgow, UK

**Keywords:** Fragility fracture, Collar and cuff, Sling, Conservative, Rehabilitation

## Abstract

**Background:**

Proximal humerus fractures (PHFs) are among the commonest bony injuries and the majority of them can be managed non-surgically. The aim of our systematic review and meta-analysis was to compare the effectiveness and safety of early versus delayed mobilisation in conservatively treated PHFs.

**Methods:**

A literature search was performed in Medline, EMBASE and clinicaltrials.gov in Januray 2025 aiming to identify all randomised controlled trials (RCTs) comparing early versus delayed (conventional) mobilisation as part of the non-surgical management of PHFs. Primary outcomes were patient-reported function and pain at short-term (3 months), mid-term (6 months) and long-term (12 months) follow-up, and secondary outcomes included secondary fracture displacement and total complications. Meta-analyses produced mean differences (MDs) or standardised MDs (SMDs) for continuous outcomes and odds ratios (ORs) for binary outcomes, with 95% confidence intervals (CI). Certainty of evidence was assessed using the GRADE tool. Recommendations for clinical practice were given only based on results of high or moderate certainty of evidence.

**Results:**

Six (6) RCTs were included that compared early mobilisation (EM; within one week from injury) to delayed mobilisation (DM; after 3 or 4 weeks of immobilisation) with a total of 470 patients with PHFs. There were no differences in patient-reported function (combined or Constant score) or pain between the EM and DM groups at any follow-up time points except for a significant difference in combined function scores favouring EM [SMD 0.4 CI (0.1,0.7), *P* = 0.006] at 3 months follow-up. There were no significant differences in the incidence of secondary fracture displacement and total complications in the two groups [OR 3.5 CI (0.7,18.2), *P* > 0.05, and OR 1.2 CI (0.5,2.9), *P* > 0.05, respectively]. All results were based on moderate or high strength of evidence. The most significant limitations of our study were the small number of pooled studies and inability to perform subgroup analyses for specific fracture types.

**Conclusions:**

Our meta-analysis of RCTs showed that commencement of mobilisation within one week from injury for non-surgically managed PHFs is safe and may confer short-term functional benefits compared to delayed mobilisation.

**Supplementary Information:**

The online version contains supplementary material available at 10.1186/s12891-025-08371-y.

## Background

Proximal humeral fractures (PHFs) are a common injury accounting for 4–10% of all fractures [1]. The majority of these fractures result from low energy trauma in elderly patients affected by osteoporosis and are referred to as fragility fractures [2, 3]. Fragility fractures are associated with significant pain, long term disability and even mortality and confer a significant healthcare cost burden; in the UK osteoporotic fractures account for approximately 2.4% of healthcare spending [4]. This represents both the direct cost of management of the initial injury and ongoing costs resulting from long-term pain, disability and need for further interventions [4]. Given that ageing is a significant risk factor for fragility fractures and that the population aged 50 years or more is projected to increase by 13.2%, the numbers and healthcare burden associated with fragility fractures is likely to increase [4].

PHFs are commonly classified based on the Neer’s classification system which describes six distinct groups based on anatomical segments and the degree of fracture displacement [5]. The management of PHFs in adults largely depends on a combination of fracture characteristics and patient factors. The management options range from non-operative interventions, reconstruction (surgical fixation), and arthroplasty (prosthetic replacement) [6]. There is great variation in practice internationally, however conservative treatment is generally accepted as the treatment option for minimally displaced fractures and displaced fractures in elderly patients whereas surgery is reserved for unstable and displaced fractures in younger patients [7]. The controversial ProFHER study demonstrated no benefits of surgery compared to non-surgical management for displaced PHFs and it has created significant debate surrounding the benefits conferred by and the indications for surgical intervention [8]. Furthermore, a recent systematic review was in agreement with the findings of the ProFHER study and additionally showed that surgical interventions for PHFs may also increase the need for further surgery [7, 8]. Conservative treatment generally consists of analgesia and a period of immobilisation in a sling followed by a rehabilitation programme for the patient to regain movement and strength [7].

Definitive guidelines on the duration of immobilisation and timing of commencement of the rehabilitation programme do not exist. In theory, mobilisation that starts too early may risk secondary fracture displacement, whilst prolonged immobilisation may lead to long-term stiffness. Given that the vast majority of PHFs are managed non-surgically and their increasing incidence, more focus should be placed on optimising non-surgical management both from a patient and healthcare system perspectives.

The aim of our systematic review and meta-analysis was to compare the effectiveness and safety of early mobilisation versus conventional mobilisation as part of the non-surgical management of PHFs.

## Methods

This systematic review and meta-analysis was prospectively registered on PROSPERO (CRD42023395162), conducted and authored as per PRISMA guidance.

### Eligibility criteria

#### Types of studies

Randomised Control Trials (RCTs) of any type were eligible for inclusion. Only studies published in English were screened for inclusion.

#### Types of participants

Studies of patients with acute PHFs were eligible for inclusion if they were managed non-surgically. A radiographic diagnosis by a radiologist or orthopaedic surgeon and management and follow-up in an orthopaedic/fracture clinic were necessary. Type of fracture and amount of radiographic displacement were not criteria and neither was duration or regime of post-injury mobilisation/physiotherapy, however they were taken into account for certainty of evidence assessment in the “indirectness” (clinical heterogeneity) domain. The present study excluded trials with (1) patients < 18 years of age, (2) patients that were treated surgically.

#### Types of interventions

Early mobilisation (EM; commencing within one week from injury), supervised or unsupervised.

#### Types of comparators

“Delayed” mobilisation after at least 3 weeks of immobilisation in a sling/brace (DM; conventional treatment/control).

#### Types of outcome measures

The primary outcomes were patient-reported function measured by any scale and patient-reported pain measured by the visual analogue scale (VAS) or equivalent (0–10 or 0-100). Secondary outcomes included secondary fracture displacement and total complications. For the purpose of analysis and pooling of results, outcome measures were divided into three distinct intervals, short-term (≤ 12 weeks), mid-term (> 12 weeks – <12 months) and long-term (**≥** 12 months). Where studies reported results at more than one time points within our pre-specified intervals, those closest to the later end of the interval were used.

When trials used different concepts to assess patient-reported pain, the following hierarchy was used: (a) pain at rest, (b) pain with (any) activity, (c) tenderness.

### Literature search

Search strategies were developed by two authors independently in “all fields” with relevant Boolean operators and their combinations in EMBASE, Medline and clinicaltrials.gov databases in January 2025. The search strategies are shown in Suppl. Figure 1.

The reference and citation lists of all eligible studies were screened for further eligible trials. The PRISMA flowchart is illustrated in Suppl. Figure 2. A total of 315 studies were identified after removal of duplicates across all databases. After removal of non-eligible study types and non-relevant studies, the abstracts and full texts of the remaining studies were screened for inclusion by two authors independently.

### Data extraction

Patient characteristics, characterisation of included fractures, duration of immobilisation prior to commencement of mobilisation, brace type, outcome measures, follow-up time points and follow-up completion data were extracted from individual trials and recorded in Microsoft Word version 16.43 (Microsoft corp) by two authors separately (DC, HM) in a previously constructed data extraction table. Disagreements were resolved with the involvement of the senior author (NLM). For missing data, attempts were made to contact the corresponding authors of studies published in the last 5 years.

### Data handling – synthesis of results

Results of the same outcome reported by two or more studies at the same pre-specified follow-up time points (short-term, mid-term, long-term) were pooled quantitatively by pairwise meta-analyses in the absence of significant clinical heterogeneity (similar populations, follow-up time points and interventions). The appropriateness of pooling in the presence of some clinical heterogeneity was a joint decision between the first and senior authors (DC, NLM). Raw mean differences (MD) and odds ratios (OR) with their accompanying 95% confidence interval (CI) were calculated and used in the tests for continuous and dichotomous outcomes respectively. Where different scales assessing the same outcome (e.g., Constant score and Simple Shoulder Test) were pooled in meta-analyses, standardised mean differences (SMDs) were calculated instead of raw MDs. Statistical heterogeneity was calculated with the I^2^ statistic for each meta-analysis.

### Risk of bias and strength of evidence assessment

The Cochrane Risk of Bias Tool 2 (RoB 2) was used to assess risk of bias for each RCT [9]. Studies were assessed by two authors (DC, HM) separately and controversies were resolved with involvement of the senior author (NLM). The overall RoB for each RCT was labelled as “low”, “some concerns” or “high” based on the result of the tool’s algorithm and the assessor’s judgment.

The Grading of Recommendations Assessment, Development and Evaluation (GRADE) was used to appraise the certainty (strength) of evidence [10]. For each pairwise meta-analysis, certainty of evidence for its result was assessed based on five domains: overall RoB, inconsistency, imprecision, indirectness and other confounding factors (including publication bias). The result of each comparison of interventions was assigned one of high, moderate, low or very low certainty of evidence. This process was completed independently by two authors (DC, HM) for each outcome measure and disagreements were resolved by involvement of the senior author (NLM). The certainty of evidence started from “high” and was downgraded for one step each time when any one of the five domains raised concerns; Where statistical heterogeneity (“inconsistency”) was found to be high (I^2^ **=** 50–79%), the certainty of evidence was downgraded by one level; where it was found to be substantial (I^2^ ≥ 80%), it was downgraded by two levels. In those cases, subgroup analyses were performed to try and explain this heterogeneity only when there were significant differences in populations, interventions, comparators or outcomes (PICOs) of the pooled studies. For “imprecision”, the certainty of evidence was downgraded where the optimal information size was not reached (see “statistical analysis” section) or if the 95% CI was very wide. For indirectness, the certainty of evidence was downgraded where there was clinical heterogeneity regarding any of the PICOs. Publication bias was assessed only where 10 or more studies were pooled. The certainty of evidence was upgraded when the magnitude of effect was large with both clinical and statistical significance.

Strong recommendations for clinical practice were given only based on results of high or moderate certainty of evidence. One intervention was considered more effective than the other for any outcome measure where their differences were significant both at statistical and clinical significance (> minimum clinically important difference, MCID). MCIDs were defined as follows based on previous relevant literature: (a) pain VAS = 1.4 points; (b) Constant score = 10 points; and (c) Disabilities of the Arm, Shoulder and Hand (DASH) scale = 11 points [11–13].

The Constant score (Constant-Murley score) is a 100-point scale that aims to assess function of the shoulder. Two of its subscales are patient-reported (pain and activities of daily living) and the other two are measured by assessors (strength and range of movement). The DASH scale is a 30-item, 100-point questionnaire that aims to assess functional disability due to upper extremity disorders through activities of daily living and is exclusively patient-reported.

### Statistical analysis

The Review Manager V.5 (RevMan) software was used to calculate pooled MDs, SMDs and ORs and generate forest plots for pairwise meta-analyses and their accompanying statistical heterogeneity tests (Chi^2^ and I^2^). Where SMDs were calculated in meta-analyses for function, these were converted to MDs for each participating function scale (Constant score and DASH) to assess for clinical significance.

The following formula was used for the sample size calculation as part of GRADE’s assessment for imprecision:

$$\:N=\:\frac{2{(a+b)}^{2}{SD}^{2}}{{\text{x}}^{2}}$$

Where:

N = the sample size required in each of the groups (optimal information size).

x = MCID.

SD^2^ = population variance (calculated using pooled SD from included treatment groups, 1.6 points for VAS, 17.1 points for Constant score, 14.8 points for DASH scale).

a = 1.96 (for 5% type I error).

b = 0.842 (for 80% power).

With the use of the above formula, the optimal information size (minimum number of overall patients combined in each meta-analysis for sufficient “precision” in the GRADE assessment) was calculated as *n* = 21 patients for pain VAS, *n* = 46 for the Constant score and *n* = 29 for the DASH scale.

Potential publication bias was not assessed as no pairwise meta-analyses included more than 10 studies. Expecting wide-range variability in studies’ settings, a random-effects model was employed in all meta-analyses.

### Protocol deviations

“Quality of life” and “active ROM” were removed from our secondary outcome measures as they were only included in single RCTs and could not be pooled; instead, we included total complications as a secondary outcome as we thought it would be an important consideration for practice recommendations.

## Results

A total of 6 studies were found to be eligible and were included in meta-analyses, with a total of 470 patients with PHFs [14–18, 19]. Table 1 summarises the relevant characteristics of these studies. Two studies were assessed as being of overall “high” and three of “low” RoB and one raised “some concerns” (Suppl. Table 1). Included patients in all studies had PHFs fractures for which non-surgical management was deemed appropriate by the treating orthopaedic team. Descriptions of these varied among studies (Table 1). Three studies immobilised patients in the EM group for 1 week and the other three commenced immediate mobilisation. Five [5] studies immobilised patients in the DM group for 3 weeks and one for 4 weeks. Three studies used a sling bandage for immobilisation, one used collar and cuff, one sling and body bandage and this information was not available in two studies. For these two studies, we obtained unpublished data from a recent systematic review by Handoll et al. (2022) who contacted the researchers of the studies which were terminated early [20]. Mobilisation regimes were similar in all studies, starting with pendulum exercises and then progressing gradually to passive and active mobilisation either with or without physiotherapist supervision. Final follow-up time points in the 6 studies ranged from 6 to 24 months. Suppl. Tables 2–6 summarise the findings of the included studies.

**Table 1:** Characteristics of the included randomised controlled trials.* DASH*,* disabilities of arm*,* shoulder and hand; EQ-5D*,* EuroQoL 5 dimensional; ER*,* external rotation; IR*,* internal rotation; M*,* weeks; QoL*,* quality of life; VAS*,* visual analogue scale; w*,* weeks*

Study	Fractures included	Population size (mean age, %F); Interventions	Outcome Measures	Follow up time points	Immobilisation method	% follow up completion
Martinez et al. (2021)	“>50% contact between diaphysis and humeral head (1-, 2-, 3-, 4-part)”	*N* = 143 (mean 70y, 79% F) Immobilisation for 1 week *n* = 67 Immobilisation for 3 weeks *n* = 76	Pain VAS, Constant score (function), Simple Shoulder Test (function), secondary displacement at 1w and 3w, non-union at 6 m, osteonecrosis 12 m and 24 m, complications	3w, 3 m, 6 m, 12 m, 24 m	Sling bandage	77.6%
Lefevre-Colau et al. (2007)	“Impacted fracture of epiphysis or metaphysis (1-, 2- and 3-part)”	*N* = 74 (mean 63y, 73% F) Early mobilisation (< 72 h from injury) *n* = 37 Immobilisation for 3 weeks *n* = 37	Pain VAS, Constant score (function), active and passive ROM (compared to contralateral shoulder), satisfaction, fracture displacement, fracture union	6w, 3 m, 6 m	Sling bandage	86.5%
Hodgson et al. (2003)	“Minimally displaced two-part fracture, including those of the greater tuberosity”	*N* = 86 (mean 70y, 81% F) Immediate physiotherapy *n* = 44 Immobilisation for 3 weeks *n* = 42	Constant score (function), SF-36 questionnaire (QoL), Croft shoulder disability questionnaire	8w, 16w, 52w, 2y	Collar and cuff	95% 1y, 86% 2y
Kristiansen et al. (1989)	“Displaced 2-part, 3-part or 4-part fractures, 74% minimally displaced”	*N* = 85 (mean 72y, 71% F) Immobilisation for 1 week *n* = 42 Immobilisation for 3 weeks *n* = 43	Modified Neer score system (pain, function, mobility)	1 m, 3 m, 6 m, 1y, 2y	Sling and body bandage	86% 1y, 46% 2y
Ring et al. (2019)	“Minimal displacement”	*N* = 63 (mean 63y, 28% F) Immediate physiotherapy *n* = 26 Immobilisation for 3 weeks *n* = 24	DASH (function), shoulder flexion, shoulder pain, ER and IR, abduction, mortality, serious and other adverse effects	6 m	Not available	48% 6 m
Torrens et al. (2012)	“Displaced (2-part or 3-part) or non-displaced fractures that were either not considered for surgery or patient refused surgery”	*N* = 42 (mean 70y, 76% F) Immobilisation for 1 week *n* = 20 Immobilisation for 4 weeks *n* = 22	Pain VAS, Constant score (function), Satisfaction VAS, EQ-5D (QoL), mortality, secondary surgery and complications, significant displacement	1w, 3w, 3 m, 6 m, 12 m	Not available	95%

**Fig. 1:**
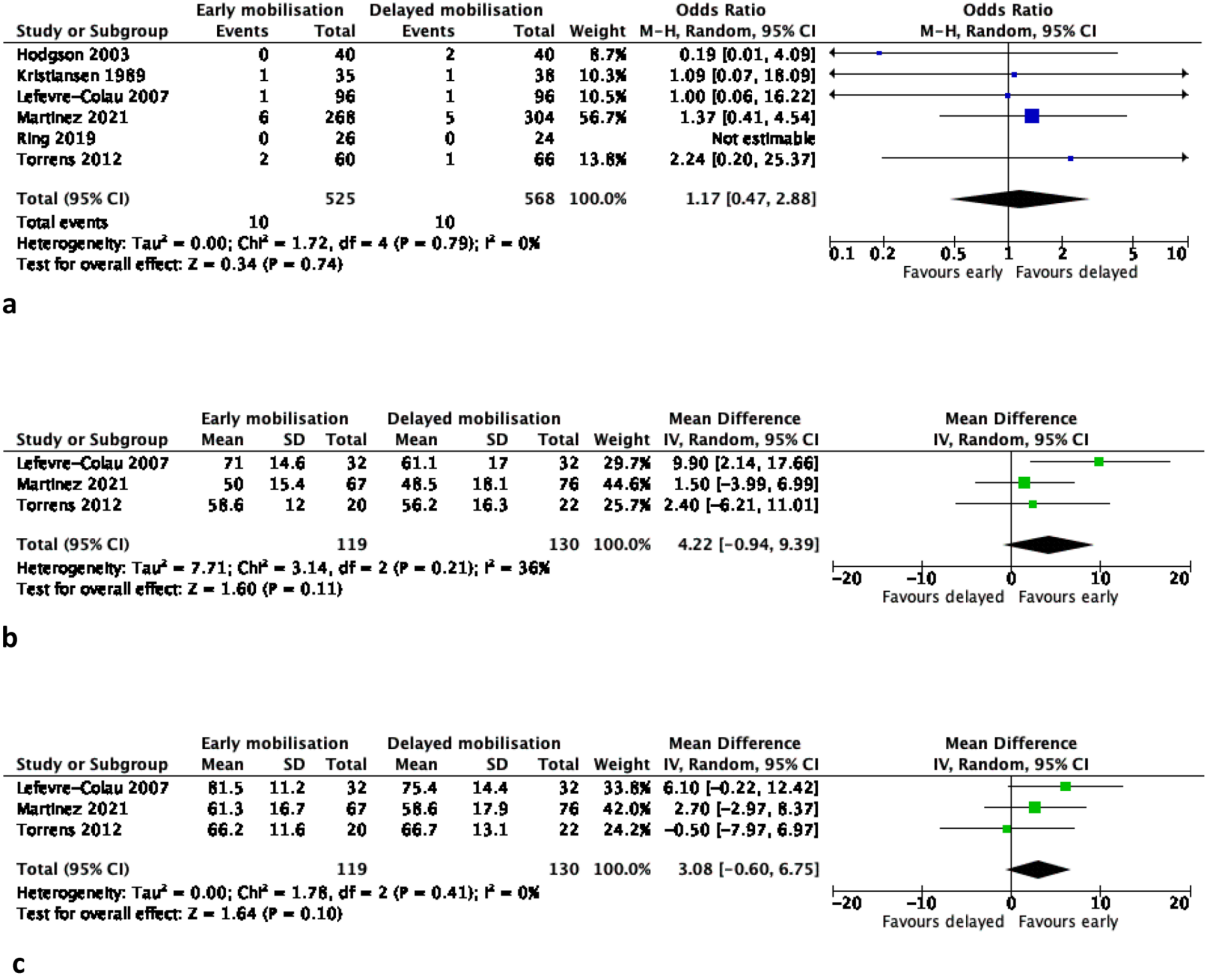
**(a-c)**: Meta-analysis results of patient-reported function (all scales) at 3 months (1a), 6 months (1b) and 12 months (1c) for early versus delayed mobilisation of non-surgically treated proximal humerus fractures. The pooled results are expressed as a standardised mean difference (SMD) with 95% confidence interval, p value and statistical heterogeneity tests (Chi^2^ and I^2^)

**Fig. 2:**
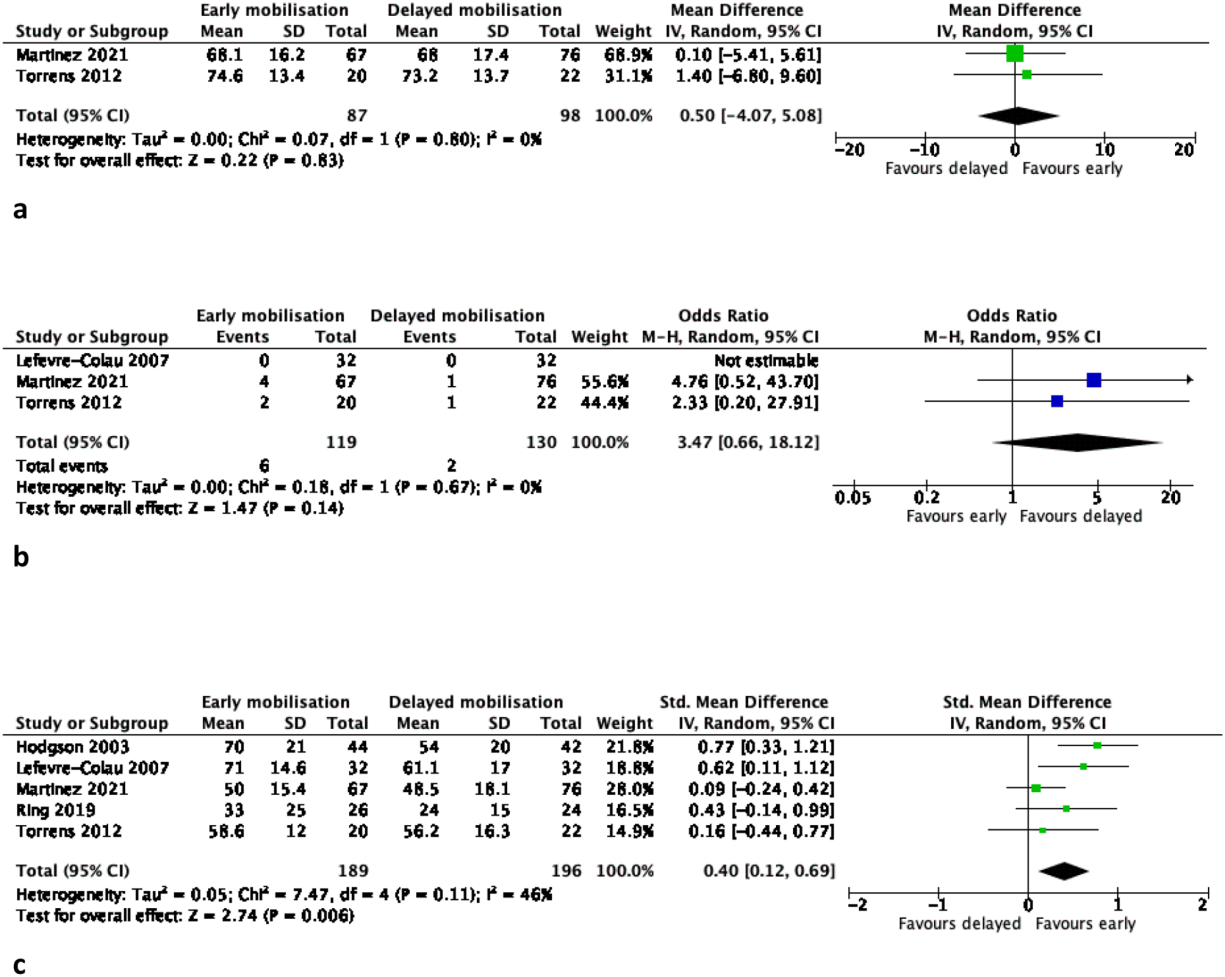
**(a-c)**: Meta-analysis results of patient-reported Constant score (function) at 3 months (2a), 6 months (2b) and 12 months (2c) for early versus delayed mobilisation of non-surgically treated proximal humerus fractures. The pooled results are expressed as a raw mean difference (MD) with 95% confidence interval, p value and statistical heterogeneity tests (Chi^2^ and I^2^)

**Fig. 3:**
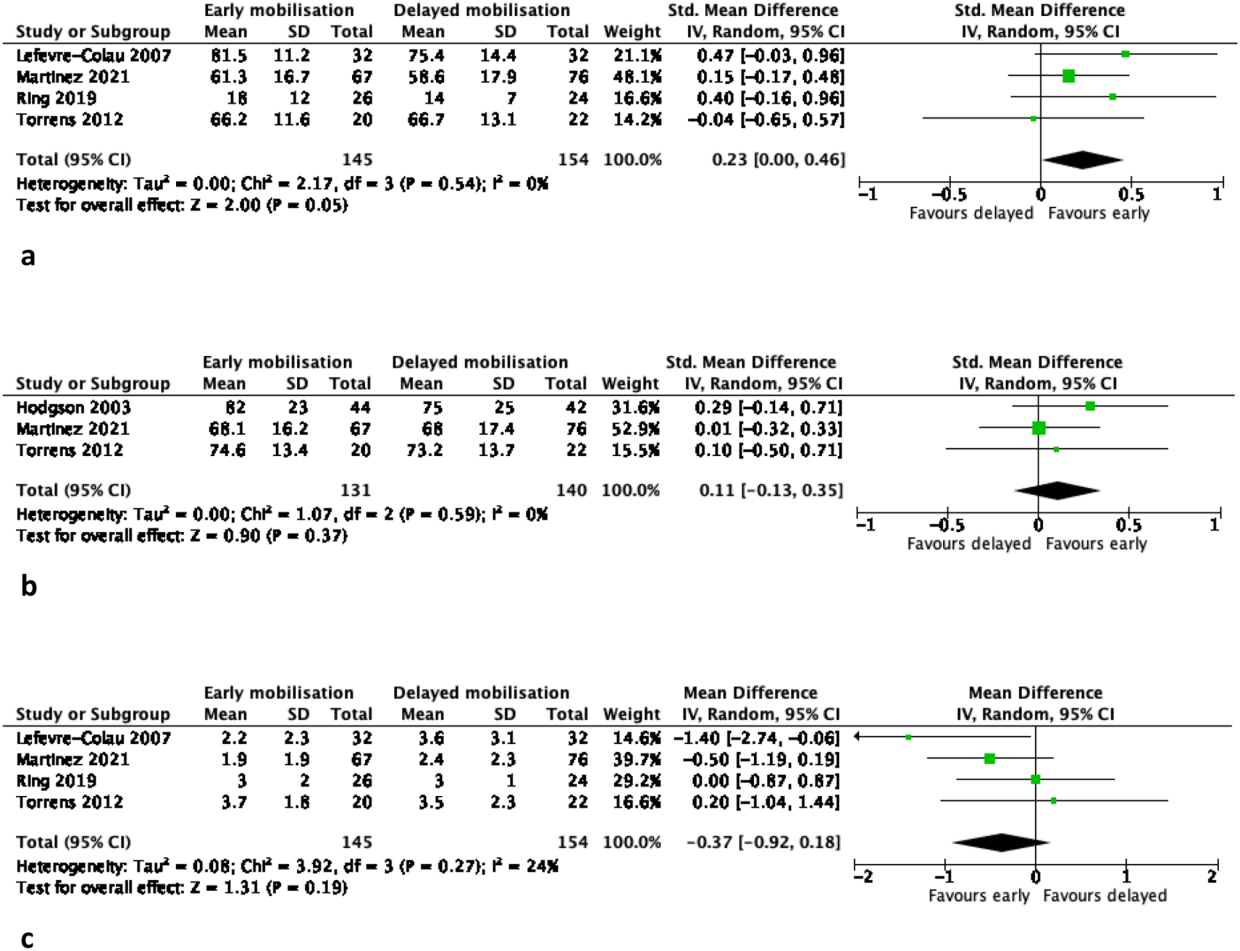
**(a-c)**: Meta-analysis results of patient-reported pain (visual analogue scale) at 3 months (3a), 6 months (3b) and 12 months (3c) for early versus delayed mobilisation of non-surgically treated proximal humerus fractures. The pooled results are expressed as a raw mean difference (MD) with 95% confidence interval, p value and statistical heterogeneity tests (Chi^2^ and I^2^)

**Fig. 4:**
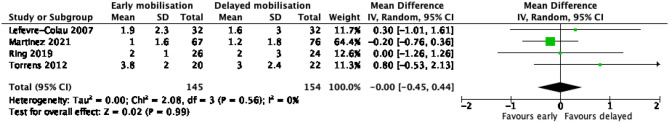
Meta-analysis results of secondary fracture displacement for early versus delayed mobilisation of non-surgically treated proximal humerus fractures. The pooled result is expressed as odds ratio (OR) with 95% confidence interval, p value and statistical heterogeneity tests (Chi^2^ and I^2^)

**Fig. 5:**
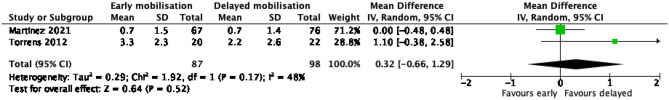
Meta-analysis results of total complications for early versus delayed mobilisation of non-surgically treated proximal humerus fractures. The pooled result is expressed as odds ratio (OR) with 95% confidence interval, p value and statistical heterogeneity tests (Chi^2^ and I^2^)

Pairwise meta-analyses comparing EM versus DM in non-surgically treated PHFs produced the following results (Table 2; Figs. 1, 2, 3, 4 and 5):

**Table 2:** Meta-analysis results and certainty of evidence assessment using the GRADE tool. Positive values for function and negative for pain favour early mobilisation; OR below 1 favour early mobilisation

Outcome	Follow up	Pooled studies (total population)	Meta-analysis result	Overall RoB	Imprecision	Inconsistency	Indirectness	Other	Certainty of Evidence
Function – All	ST – 3 m	5 (*n* = 385)	**SMD 0.4 (0.1**,**0.7)**	Low	Low	Low	Low	Low	⊕⊕⊕⊕ High
	MT – 6 m	4 (*n* = 299)	SMD 0.2 (0.0,0.5)	High	Low	Low	Low	Low	⊕⊕⊕∅ Moderate
	LT – 12 m	3 (*n* = 271)	SMD 0.1 (-0.1,0.4)	High	Low	Low	Low	Low	⊕⊕⊕∅ Moderate
Function - Constant score	ST – 3 m	3 (*n* = 249)	MD 4.2 (-0.9,9.4)	Low	Low	Low	Low	Low	⊕⊕⊕⊕ High
	MT – 6 m	3 (*n* = 249)	MD 3.1 (-0.6,6.8)	Low	Low	Low	Low	Low	⊕⊕⊕⊕ High
	LT – 12 m	2 (*n* = 185)	MD 0.5 (-4.1,5.1)	Low	Low	Low	Low	Low	⊕⊕⊕⊕ High
Pain VAS	ST – 3 m	4 (*n* = 299)	MD -0.4 (-0.9,0.2)	High	Low	Low	Low	Low	⊕⊕⊕∅ Moderate
	MT – 6 m	4 (*n* = 299)	MD 0 (-0.5,0.4)	High	Low	Low	Low	Low	⊕⊕⊕∅ Moderate
	LT – 12 m	2 (*n* = 185)	MD 0.3 (-0.7,1.3)	Low	Low	Low	Low	Low	⊕⊕⊕⊕ High
Secondary displacement	-	3 (*n* = 249)	OR 3.5 (0.7,18.2)	Low	High*	Low	Low	Low	⊕⊕⊕∅ Moderate
Total complications	-	6 (*n* = 470)	OR 1.2 (0.5,2.9)	High	Low	Low	Low	Low	⊕⊕⊕∅ Moderate

*wide CI

*RoB*,* risk of bias*

### Function (all outcomes combined; Fig. 1)

#### Short-term follow-up (3 months)

the EM group had significantly higher patient-reported scores than the DM group (SMD 0.4 CI (0.1,0.7), *P* = 0.006, 5 studies, *n* = 385 patients, high certainty of evidence) at statistical significance. When the SMD was converted to MDs for Constant and DASH scores to assess for clinical significance, neither of them exceeded their MCID.

#### Mid-term follow-up (6 months)

no statistically significant differences between the two groups (SMD 0.2 CI (0,0.5), *P* = 0.05, 4 studies, *n* = 299 patients, moderate certainty of evidence). When the SMD was converted to MDs for Constant and DASH scores to assess for clinical significance, neither of them exceeded their MCID.

#### Long-term follow-up (12 months)

no statistically significant differences between the two groups (SMD 0.1 CI (-0.1,0.4), *P* = 0.37, 3 studies, *n* = 71 patients, moderate certainty of evidence). When the SMD was converted to MDs for Constant and DASH scores to assess for clinical significance, neither of them exceeded their MCID.

### Function – constant score (Fig. 2)

#### Short-term follow-up (3 months)

No statistically or clinically significant differences between the two groups (MD 4.2 points CI (-0.9,9.4), *P* = 0.11, 3 RCTs, *n* = 249 patients, high certainty of evidence).

#### Mid-term follow-up (6 months)

No statistically or clinically significant differences between the two groups (MD 3.1 points CI (-0.6,6.8), *P* = 0.10, 3 RCTs, *n* = 249 patients, high certainty of evidence).

#### Long-term follow-up (12 months)

No statistically or clinically significant differences between the two groups (MD 0.5 points CI (-4.1,5,1), *P* = 0.83, 2 RCTs, *n* = 185 patients, high certainty of evidence).

### Pain VAS (Fig. 3)

#### Short-term follow-up (3 months)

No statistically or clinically significant differences between the two groups (MD -0.4 points CI (-0.9,0.2), *P* = 0.19, 4 RCTs, *n* = 299 patients, moderate certainty of evidence).

#### Mid-term follow-up (6 months)

No statistically or clinically significant differences between the two groups (MD 0 points CI (-0.5,0.4), *P* = 0.99, 4 RCTs, *n* = 299 patients, moderate certainty of evidence).

#### Long-term follow-up (12 months)

No statistically or clinically significant differences between the two groups (MD 0.3 points CI (-0.7,1.3), *P* = 0.52, 2 RCTs, *n* = 185 patients, high certainty of evidence).

### Secondary fracture displacement (Fig. 4)

There was no statistically significant difference in the incidence of fracture displacement at follow-up (OR 3.5 CI (0.7,18.2), *P* = 0.14, 3 studies, *n* = 249 patients, moderate certainty of evidence).

### Total complications (Fig. 5)

There was no statistically significant difference in the incidence of all complications at follow-up (OR 1.2 CI (0.5,2.9), *P* = 0.74, 6 studies, *n* = 470 patients, moderate certainty of evidence).

## Discussion

In the present meta-analysis, we demonstrated that immediate mobilisation (starting within a week from injury) was associated with similar pain VAS and Constant score compared to conventional mobilisation (starting after at least 3 weeks of immobilisation) at 3, 6 and 12 months of follow-up. Based on our analyses that combined all included function instruments, there may be short-term benefits in favour of early mobilisation, which may be clinically irrelevant, however. No significant differences were identified for secondary fracture displacement or total complications. All our findings were based on moderate or high certainty of evidence, which suggests that the results are close to the true effects and further research would be rather unlikely to change these results.

The management of PHFs remains controversial. Indications for surgical interventions remain poorly defined and when a decision for surgery is made, the type of surgery (fixation versus arthroplasty) is also contentious [21, 22]. Even for arthroplasty, options include hemiarthroplasty or reverse polarity total shoulder arthroplasty (RTSA) [23, 24]. The ProFHER study, a pragmatic multi-centre randomised trial, found no clinical differences at any follow-up time point up to 2 years in those with displaced PHFs treated surgically and non-surgically [8]. Non-surgical management also had favourable cost implications in the UK healthcare setting. The ProFHER 2 study is currently ongoing and its aim is to compare hemiarthroplasty with RTSA along with the effectiveness of either compared with non-surgical management in patients over 65 with PHFs [25].

When non-surgical management is chosen for these injuries, the conventional immobilisation period is at least 3 weeks and the rationale for this is to allow for some early fracture consolidation which will make the fracture more stable before mobilisation commences. This is not, however based on evidence. None of the included RCTs showed an increase risk of secondary displacement with early mobilisation. The incidence of this in the largest of our included RCTs by Martinez et al. (2021) was 4 in the EM group versus 1 in the DM group however due to these values being very small this was not statistically significant [17]. Only one of the 4 patients required further treatment, in the form of surgical fixation. A previous retrospective study by Aguado et al. (2018), in which all patients with PHFs were treated with early mobilisation, found that radiographically the shaft displaced into a more optimal position soon after the injury, however there was a rather significant incidence (around 20%) for greater tuberosity fragments to displace superiorly [26]. Although the authors are attributing these effects to the exercise programme, these effects may well be the natural progression of the fracture regardless of rehabilitation.

Risk factors for secondary fracture displacement in conservatively treated PHFs have previously been studied. For isolated greater tuberosity fractures, Brockmann et al. (2019) retrospectively compared fractures that did displace and those that did not and found that important risk factors for displacement were older age, previous shoulder dislocations, and split-type fractures (as described by Mutch et al. (2014) [27]. This study is partly in agreement with an earlier retrospective study by Hebert-Davies et al. (2015) who showed that greater tuberosity fractures displaced 5.6 times more in those who had a fracture dislocation than those who only had a fracture [28]. However, they found that the risk of secondary displacement was higher in younger patients. A similar retrospective study looking at all types of PHFs by Frank et al. (2020) found alcohol abuse, previously diagnosed osteoporosis, eccentric head index (offset of humeral head centre in relation to diaphysis) and disruption of medial hinge to be important risk factors for secondary displacement [29]. Finally, Foruria et al. (2011) found that lateral impaction (valgus) fractures had worse clinical outcomes than other types of fractures [30]. Displacement of a fractured greater tuberosity medially and of the articular surface inferiorly in posteromedial impaction fractures were also correlated with worse outcomes.

Previous reviews were in support of our findings. A recent Cochrane review by Handoll et al. (2021) found no significant differences between the EM and DM groups for self-reported shoulder function (DASH and Croft scores), QoL, mortality and adverse events; however all of these results were based on very low certainty of evidence as the majority of them were based on single studies. A similar systematic review by Ostergaard et al. (2021) reported the same findings as the Cochrane review with low or very low certainty of evidence. The maximum number of pooled studies was two and some of their results were presented qualitatively only. Since the publication of these two systematic reviews, an additional RCT has been published, which was used for pooling in our meta-analyses and substantially increased the certainty of evidence [17]. Additionally, we pooled studies reporting results of any functional instrument and reported SMDs and we used the more recent version of the Cochrane RoB tool.

Definitive guidelines on rehabilitation regimes regarding their timing, intensity, frequency or duration do not exist. In their randomised study, Carbone et al. (2017) compared early intensive mobilisation to early conventional mobilisation in patients treated non-surgically for osteoporotic PHFs [31]. Both groups had a sling on for 3 weeks and were allowed to remove it for the exercises and the regime was the same in the two groups, however the intensive mobilisation group performed exercises 5 times a week and the conventional group twice a week for the first 10 sessions. The functional outcome measures were similar in the two groups at all follow-up time points (up to 12 months) and no fracture lost its reduction radiographically in either group. In another RCT by Tousignant et al. (2020) telerehabilitation using a video conferencing system was found to be as effective as face-to-face physiotherapy with regard to ROM, function and satisfaction [32]. Finally, self-exercise was demonstrated to be as effective as face-to-face physiotherapy for the rehabilitation of non-surgically treated PHFs [33].

We do recognise the limitations of our study. There was only a small number of eligible RCTs available for inclusion and their description of the included PHFs varied, which however reflects the variability encountered in clinical practice. Despite the small number of pooled studies, the certainty of evidence of all our results was moderate or high and this is thanks to minimal statistical heterogeneity, generally narrow confidence intervals, adequate optimal information sizes of the pooled populations and generally low overall RoB. We used the RoB 2 and GRADE tools for RoB and certainty of evidence assessment respectively, which are the recommended tools for systematic reviews of RCTs by the Cochrane collaboration, and only pooled results at similar follow-up time points. Subgroup analyses for specific fracture types were not possible as the included studies did not report data separately for each fracture type. Finally, although clinical heterogeneity was not significant enough to preclude pooling of studies or downgrading the certainty of evidence, the minor differences in fracture types, duration and method of immobilisation and rehabilitation protocols may have influenced the pooled estimates.

Based on our results and the relevant literature, we recommend commencement of early rehabilitation for non-surgically managed PHFs. The early pendulum exercises will minimise stiffness, they are safe with regard to secondary fracture displacement whilst it has been postulated that they may even improve fracture alignment [26]. A sling or collar and cuff should be provided from the outset, however it should be made clear to patients that it is purely for their own comfort and they can decide when and how much to wear it in the first few weeks. Finally, greater tuberosity fractures, either isolated or alongside surgical neck fractures, should be watched closely radiographically for at least 2 weeks after the injury as early surgical intervention would be indicated if they displace significantly. Their presence should not influence the use of sling or timing of commencement of rehabilitation, however. Risk factors for fracture re-displacement remain unclear and whether early mobilisation increases the risk of re-displacement in the presence of those risk factors is also uncertain. Delayed mobilisation for selected cases (e.g. greater tuberosity fractures associated with dislocations, unreliable patients, etc.) should be at the discretion of the treating physician and should be judged on a case-by-case basis.

## Conclusion

Our meta-analysis of RCTs showed that commencement of rehabilitation within a week of injury is safe for conservatively managed PHFs, and it may be associated with some short-term functional benefits. Long-term function, pain and total complications were the same in the early and conventional mobilisation groups. Treating clinicians should consider the purpose of prolonged periods of immobilisation as we have demonstrated no clinical benefits and it can be restrictive and inconvenient for patients. Similar work should be done on other conservatively managed injuries, such as distal radius and ankle fractures, and future studies should focus on the early prediction of secondary displacement of PHFs.

## Electronic supplementary material

Below is the link to the electronic supplementary material.

Supplementary Material 1

## Data Availability

The datasets used and/or analysed during the current study are available from the corresponding author upon reasonable request.
