# Supplementary material for: Early versus delayed mobilisation for non-surgically treated proximal humerus fractures: a systematic review and meta-analysis of randomised trials
Source: BMC Musculoskelet Disord. 2025 Feb 27;26:203. doi: 10.1186/s12891-025-08371-y (PMC11866563; doi:10.1186/s12891-025-08371-y)
Supplement: Supplementary file 1 — Supplementary Material 1 [file 12891_2025_8371_MOESM1_ESM.docx]

| **Studies** | Domain 1 | Domain 2 | Domain 3 | Domain 4 | Domain 5 | Overall RoB |
| --- | --- | --- | --- | --- | --- | --- |
|  | Randomisation process | Deviations from intended interventions | Missing outcome data | Measurement of the outcome | Selection of the reported result |  |
| **Martinez et al. (2021) (n=67vs76)** | Low | Low | Low | Some concerns | Low | Low |
| **Ring et al. (2019) (n=26vs24)** | Low | Low | High | Some concerns | Some concerns | High |
| **Torrens et al. (2012) (n=20vs22)** | Some concerns | Low | Low | Some concerns | Low | Some concerns |
| **Lefevre-Colau et al. (2007) (n=32vs32)** | Low | Low | Low | Some concerns | Low | Low |
| **Hodgson et al. (2003) (n=44vs42)** | Some concerns | Low | Low | Some concerns | Low | Low |
| **Kristiansen et al. (1989) (n=42vs43)** | Some concerns | Some concerns | Low* | Some concerns | Some concerns | High |

**Supplementary Tables**

**Suppl. Table 1: Risk of Bias assessment using Cochrane’s RoB 2.0 tool**

*RoB, risk of bias*

*Up to 1y follow up only (2y follow up data not used in any meta-analyses)

| **Study** | **Scale** | **Early mobilisation** | | | | **Delayed mobilisation** | | | |
| --- | --- | --- | --- | --- | --- | --- | --- | --- | --- |
|  |  | **3m** | **6m** | **12m** | **24m** | **3m** | **6m** | **12m** | **24m** |
| **Martinez et al. (2021) (n=67vs76)** | Constant score | 50.0 (15.4) | 61.3 (16.7) | 68.1 (16.2) | 67.9 (18.8) | 48.5 (18.1) | 58.6 (17.9) | 68.0 (17.4) | 71.8 (16.2) |
| **Ring et al. (2019) (n=26vs24)** | DASH score | 33.0 (25.0) | 18.0 (12.0) | - | - | 24.0 (15.0) | 14.0 (7.0) | - | - |
| **Torrens et al. (2012) (n=20vs22)** | Constant score | 58.6 (12) | 66.2 (11.6) | 74.6 (13.4) | - | 56.2 (16.3) | 66.7 (13.1) | 73.2 (13.7) | - |
| **Lefevre-Colau et al. (2007) (n=32vs32)** | Constant score | **71.0 (14.6)** | 81.5 (11.2) | - | - | 61.1 (17.0) | 75.4 (14.4) | - | - |
| **Hodgson et al. (2003) (n=44vs42)** | Constant score (% of other shoulder) | **70.0 (21.0)** | - | 82.0 (23.0) | - | 54.0 (20.0) | - | 75.0 (25.0) | - |
| **Kristiansen et al. (1989) (n=42vs43)** | Modified Neer score | 22/30 (no SD) | 25/30 (no SD) | 25/30 (no SD) | 26/30 (no SD) | 19/30 (no SD) | 25/30 (no SD) | 26/30 (no SD) | 26/30 (no SD) |

**Suppl. Table 2: Patient-reported function results.** Bold values denote statistical superiority compared to the other group

*DASH, disabilities of arm, shoulder and hand; m, months SD, standard deviation*

| **Study** | **Scale** | **Early mobilisation** | | | | **Delayed mobilisation** | | | |
| --- | --- | --- | --- | --- | --- | --- | --- | --- | --- |
|  |  | **3m** | **6m** | **12m** | **24m** | **3m** | **6m** | **12m** | **24m** |
| **Martinez et al. (2021) (n=67vs76)** | VAS (0-10) | 1.9 (1.9) | 1.0 (1.6) | 0.7 (1.5) | 0.6 (1.4) | 2.4 (2.3) | 1.2 (1.8) | 0.7 (1.4) | 0.3 (0.8) |
| **Ring et al. (2019) (n=26vs24)** | Likert (0-10) | 3.0 (2.0) | 2.0 (1.0) | - | - | 3.0 (1.0) | 2.0 (3.0) | - | - |
| **Torrens et al. (2012) (n=20vs22)** | VAS (0-100) | 37.0 (18.1) | 38.0 (20.0) | 33.1 (23.0) | - | 35.0 (23.0) | 30.0 (24.0) | 22.3 (26.0) | - |
| **Lefevre-Colau et al. (2007) (n=32vs32)** | VAS (0-100) | **22.4 (22.9)** | 18.5 (22.9) | - | - | 35.9 (31.2) | 16.1 (29.9) | - | - |
| **Hodgson et al. (2003) (n=44vs42)** | SF-36 (0-100) | 72.0 (20.6) | - | 69.2 (27.2) | - | 59.9 (20.0) | - | 65.6 (26.6) | - |
| **Kristiansen et al. (1989) (n=42vs43)** | Modified Neer score (higher scores better) | **27/35 (no SD)** | 30/35 (no SD) | 32/35 (no SD) | 35/35 (no SD) | 20/35 (no SD) | 28/35 (no SD) | 30/35 (no SD) | 35/35 (no SD) |

**Suppl. Table 3: Patient-reported pain results.** Bold values denote statistical superiority compared to other group

*m, months SD, standard deviation; VAS, visual analogue scale.*

| **Study** | **Scale** | **Early mobilisation** | | | | **Delayed mobilisation** | | | |
| --- | --- | --- | --- | --- | --- | --- | --- | --- | --- |
|  |  | **3m** | **6m** | **12m** | **24m** | **3m** | **6m** | **12m** | **24m** |
| **Torrens et al. (2012) (n=20vs22)** | EQ-5D (0-1; higher scores better) | 0.6 (0.2) | 0.7 (0.2) | 0.7 (0.2) | - | 0.7 (0.2) | 0.7 (0.2) | 0.8 (0.2) | - |
| **Hodgson et al. (2003) (n=44vs42)** | SF-36 (0-100; average of all scores) | 73.2 (28.7) | - | 70.5 (31.1) | - | 65.7 (28.2) | - | 67.9 (31.6) | - |

**Suppl. Table 4: Patient-reported quality of life results**

EQ-5D, EuroQoL 5 dimensional; m, months; SF-36, short form 36

| Study | **Early mobilisation** | | | **Delayed mobilisation** | | | **Odds Ratio** |
| --- | --- | --- | --- | --- | --- | --- | --- |
|  | Events | Total | % | Events | Total | % |  |
| **Martinez et al. (2021) (n=67vs76)** | 4 | 67 | 5.9% | 1 | 76 | 1.3% | 4.8 (0.5-43.7) |
| **Torrens et al. (2012) (n=20vs22)** | 2 | 20 | 10.0% | 1 | 22 | 4.5% | 2.2 (0.2-22.5) |
| **Lefevre-Colau et al. (2007) (n=32vs32)** | 0 | 32 | 0% | 0 | 32 | 0% | NA |

**Suppl. Table 5:** Secondary fracture displacement

*NA, not applicable*

| Study | **Early mobilisation** | | | **Delayed mobilisation** | | | **Odds Ratio** |
| --- | --- | --- | --- | --- | --- | --- | --- |
|  | Events | Total | % | Events | Total | % |  |
| **Martinez et al. (2021) (n=67vs76)** | 6 | 268 | 2.2 | 5 | 304 | 1.6% | 1.4 (0.4-1.5) |
| **Ring et al. (2019) (n=26vs24)** | 0 | 26 | 0% | 0 | 24 | 0% | NA |
| **Torrens et al. (2012) (n=20vs22)** | 2 | 60 | 3.3% | 1 | 66 | 1.5% | 2.2 (0.2-25.4) |
| **Lefevre-Colau et al. (2007) (n=32vs32)** | 1 | 96 | 0.1% | 1 | 96 | 0.1% | 1.0 (0.1-16.2) |
| **Hodgson et al. (2003) (n=44vs42)** | 0 | 40 | 0% | 2 | 40 | 5.0% | 0.2 (0.0-4.1) |
| **Kristiansen et al. (1989) (n=42vs43)** | 1 | 35 | 2.8% | 1 | 38 | 2.6% | 1.1 (0.1-18.1) |

**Suppl. Table 6:** **Total complications** (% represents % complications per total possible events, not patients)

*NA, not applicable*

**Supplementary Figures**

**
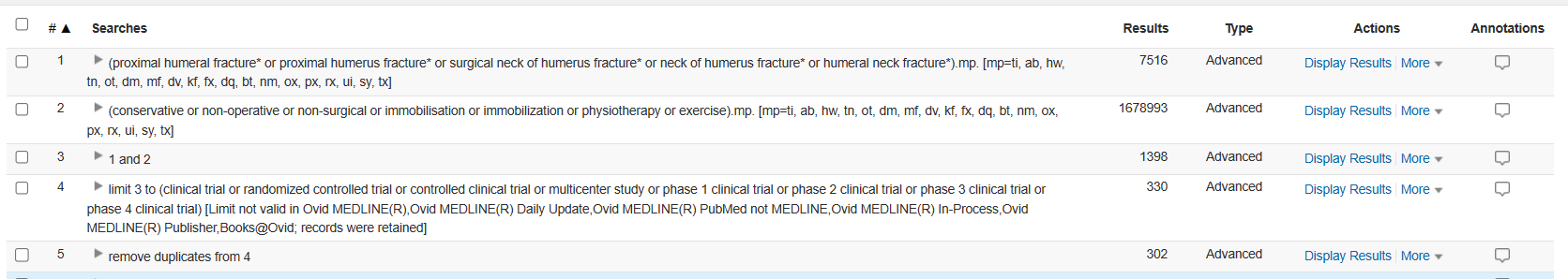
**

**1a**

**
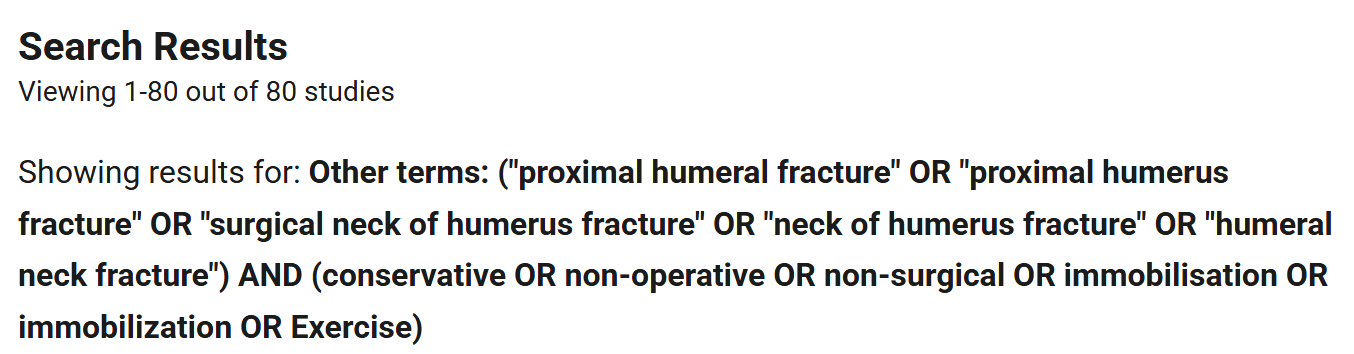
**

**1b**

**Supp. Figure 1.** **Search strategy for Medline and EMBASE (via Ovid) (1a) and clinicaltrials.gov (1b) databases**

**Identification of studies via databases**

**Identification of studies via other methods**

**Identification**

**Screening**

Studies included in qualitative and quantitative synthesis (n = 6)

**Supp. Figure 2.** **PRISMA flowchart showing the process of eligible study selection**

**Included**

**Eligibility**

Full texts excluded (n = 3)

Non-eligible interventions included (n=2), non-randomised studies (n=1)

Full texts assessed for eligibility

(n = 9)

Abstracts excluded (n = 61)

Non-randomised studies, non-English articles, reviews, topic irrelevant

Abstracts screened

(n = 70)

Titles excluded as topic irrelevant

(n = 232)

Titles screened after duplicates removed

(n = 302)

Duplicate records excluded

(n = 284)

Records identified from

Clinicaltrials.gov (n = 132)

Records identified from Medline, OVID and CENTRAL: (n = 454)
